# Women’s autonomy in refusing risky sex in sub-Saharan Africa: Evidence from 30 countries

**DOI:** 10.1371/journal.pgph.0002350

**Published:** 2023-09-14

**Authors:** Eugene Budu, Bright Opoku Ahinkorah, Joshua Okyere, Abdul-Aziz Seidu, Richard Gyan Aboagye, Sanni Yaya

**Affiliations:** 1 Korle Bu Teaching Hospital, Accra, Ghana; 2 Faculty of Health, School of Public Health, University of Technology Sydney, Sydney, Australia; 3 Department of Population and Health, University of Cape Coast, Cape Coast, Ghana; 4 Department of Nursing, College of Health Sciences, Kwame Nkrumah University of Science and Technology, Private Mail Bag, University Post Office, Kumasi, Ghana; 5 Centre for Gender and Advocacy, Takoradi Technical University, Takoradi, Ghana; 6 College of Public Health, Medical and Veterinary Sciences, James Cook University, Townsville, Australia; 7 Department of Family and Community Health, Fred N. Binka School of Public Health, University of Health and Allied Sciences, Hohoe, Ghana; 8 School of International Development and Global Studies, University of Ottawa, Ottawa, Canada; 9 The George Institute for Global Health, Imperial College London, London, United Kingdom; University of Ghana, GHANA

## Abstract

Understanding the factors associated with women’s autonomy to refuse risky sex is imperative to inform the development of policies and interventions to reduce the risk of unintended pregnancies, sexually transmitted infections, unsafe abortion, and maternal mortality. This study sought to examine the prevalence and factors associated with women’s autonomy to refuse risky sex in sub-Saharan Africa (SSA). Data for the study were extracted from the most recent Demographic and Health Surveys (DHS) of thirty countries in SSA conducted from 2010 to 2020. We included a weighted sample of 260,025 women who were married or cohabiting in the final analysis. Percentages were used to present the results of the prevalence of women’s ability to refuse risky sex. We used a multilevel logistic regression analysis to examine the factors associated with women’s ability to refuse risky sex. Stata software version 16.0 was used for the analysis. We found that 61.69% (95% confidence interval [CI]: 56.22–67.15) of the women were autonomous to refuse sex if their partners have other women, and this was highest in Namibia (91.44% [95% CI: 90.77–92.18]) and lowest in Mali (22.25% [95% CI: 21.24–23.26]). The odds of autonomy in refusing risky sex was higher among women with higher education (adjusted odds ratio [aOR] = 1.88; 95% CI = 1.78–1.46) compared to those with no formal education. Employment status was also a significant predictor, with working women having higher odds of sex refusal compared to non-working women (aOR = 1.16; 95% CI = 1.13–1.18). Advocacy to improve women’s autonomy to refuse risky sex must leverage the mass media as it emerged as a significant factor. Policies and intervention to enhance women’s autonomy must also target high-risk sub-populations which constitutes adolescent girls, those with no formal education, and those without employment.

## Introduction

Globally, unintended and mistimed pregnancies are of significant public health concern. Evidence indicates that 44% of pregnancies among women of reproductive age reported between 2010 and 2014 were unintended [1]. Corroborative to this, a Guttmacher report has revealed that nearly 121 million unintended pregnancies were reported between 2015 and 2019, of which 61% ended in abortion [2]. Evidence suggests that engaging in risky sexual behaviour is a prominent factor contributing to unintended pregnancies and abortion [3,4]. Risky sexual behaviour includes engaging in at least one of the following activities: “unprotected sexual intercourse, early initiation of sexual intercourse, multiple sexual partners, engaging in sexual intercourse under the influence of stimulant substances, or having sex immediately after watching pornographic materials” [5]. It is noteworthy that risky sexual behaviour is considered a serious public health concern due to its ability to exacerbate the risk of unintended pregnancies, unsafe abortions, sexually transmitted infections (STIs), and psychological distress [6,7].

Evidence from previous studies have shown that demographic factors such as the level of education, age, and place of residence significantly predict the likelihood of women engaging in risky sexual behaviour [8,9]. Relatedly, drunkenness or substance abuse are among the identified lifestyle behaviours that are associated with risky sexual behaviours [9,10]. Similarly, social factors such as belonging to homogenous networks and having a strong social network have been found to predict risky sexual behaviours among women [11].

Beyond these factors, the role of women’s capacity to negotiate for safe sex cannot be understated in the discussion of risky sexual behaviours. A multi-country study of 27 sub-Saharan African countries has shown that women’s capacity to negotiate for safe sex hinges on their level of autonomy [3]. Women’s autonomy refers to the capacity to make and carry out independent decisions about personal issues that are significant to their lives and those of their families [12,13]. This implies women’s freedom to take decisions concerning their health, purchases, and control over resources, as well as refusing risky sexual intercourse. Therefore, in the context of this study, women’s autonomy to refuse risky sex denotes the ability of women to freely decide not to have sex when their partner has been sexually involved with another person(s) [5].

Understanding the factors associated with women’s autonomy to refuse risky sex is imperative to inform the development of policies and interventions that would reduce the risk of unintended pregnancies, STIs, unsafe abortion, and maternal mortality; thus, contributing to the attainment of Sustainable Development Goal (SDG) 3, target 3.1 (i.e., to reduce maternal mortality to 70 per 100,000 births) and SDG 5 (achieve gender equality and empower all women and girls) [14]. For instance, a systematic review conducted in SSA has shown that women’s inability to resist risky sex contributes substantially to unintended pregnancies [15]. Despite this importance, there is limited scholarship on women’s autonomy to refuse risky sex, particularly in sub-Saharan Africa (SSA). To the best of our knowledge, there is only one study in SSA that has investigated this phenomenon [5]. However, Asabu’s study [5] was limited to only Ethiopia. As such, the findings may not be reflective of the situation in the entire SSA subregion. Indeed, Seidu et al. [3] have previously examined women’s autonomy in household decision-making and safer sex negotiation in SSA. However, that study was only concerned about whether women could or could not negotiate for safe sex. Seidu et al.’s [3] study did not explore women’s autonomy to refuse risky when the partner is in sexual relation eith other women. Thus, presenting a knowledge gap in what is known about women’s autonomy to refuse risky sex from a regional perspective in SSA. Hence, certain questions are unanswered: (a) what is the prevalence of women’s autonomy to refuse risky sex in SSA? and (b) what factors are associated with women’s autonomy to refuse risky sex in the context of SSA? The objective of this study is to estimate the prevalence and examine the factors associated with women’s autonomy to refuse risky sex in SSA. This study makes valuable contribution to literature as it shows the significant sub-populations that must be prioritised to reduce the prevalence of sexually transmitted infections resulting from engaging in risky sex.

## Materials and methods

### Data source and study design

Data for the study were extracted from the most recent Demographic and Health Surveys (DHS) of thirty countries in SSA conducted from 2010 to 2020. We pooled the data from the women’s recode files in each of the 30 countries. The DHS is a nationally representative survey conducted in over 85 low-and-middle-income countries worldwide [16]. DHS employed a descriptive cross-sectional design and the respondents were sampled using a two-stage cluster sampling method. Detailed sampling technique has been highlighted in the literature [17]. Standardized structured questionnaires were used to collect data from the respondents on health and social indicators including risky sexual behaviour [16]. We included a total of 260,025 women who were married or cohabiting in the final analysis (Table 1). The dataset used is freely available at https://dhsprogram.com/data/available-datasets.cfm. We drafted this paper with reference to the Strengthening the Reporting of Observational Studies in Epidemiology (STROBE) guidelines [18].

**Table 1:** Description of the study sample.

Country	Survey year	Weighted frequency	Weighted percentage
1. Angola	2015–16	8588	3.30
2. Burkina Faso	2010	10242	3.94
3. Benin	2017–18	9505	3.66
4. Burundi	2016–17	10573	4.07
5. Democratic Republic of Congo	2013–14	10967	4.22
6. Congo	2011–12	6239	2.40
7. Cote d’Ivorie	2011–12	5941	2.28
8. Cameroon	2018	9151	3.52
9. Ethiopia	2016	9783	3.76
10. Gabon	2012	4733	1.82
11. Ghana	2014	5556	2.14
12. Gambia	2019–20	6505	2.50
13. Guinea	2014	6440	2.48
14. Kenya	2014	18051	6.94
15. Comoros	2012	3063	1.18
16. Liberia	2019	4409	1.70
17. Lesotho	2014	3968	1.53
18. Mali	2018	6464	2.49
19. Malawi	2015–16	14876	5.72
20. Nigeria	2018	25126	9.66
21. Niger	2012	6832	2.63
22. Namibia	2013	5560	2.14
23. Rwanda	2019–20	8171	3.14
24. Sierra Leone	2019	9180	3.53
25. Senegal	2010–11	8974	3.45
26. Chad	2014–15	10326	3.97
27. Togo	2013–14	5571	2.14
28. Uganda	2016	10902	4.19
29. Zambia	2018	8248	3.17
30. Zimbabwe	2015	6076	2.34
**All countries**	**2010–2020**	**260,025**	**100.00**

### Variables

#### Outcome variable

Women’s autonomy in refusing risky sex was the outcome variable in this study. This variable was measured based on women’s response to ‘Reason for not having sex’. Wives who can refuse sex if their husbands have other women were considered as ‘autonomous in refusing risky sex’ and wives who cannot refuse sex if their husbands have other women were considered as ‘not autonomous in refusing risky sex’. For this study, the variable was then recoded into; “0” = cannot refuse risky sex and “1” = can refuse risky sex. Studies that used the DHS dataset employed similar coding and categorisation [5,19].

#### Explanatory variables

Ten explanatory variables were included in the study. These variables were selected based on their availability in the DHS dataset and their influence on women’s ability to refuse risky sex from previous studies [5,8]. These variables were grouped into individual level and contextual level variables. The individua level variables were women’s age, educational level, employment status, partner’s educational level, partner’s employment status, age at first sex, and exposure to mass media [3]. The contextual level variables were wealth index, type of place of residence, and geographical sub-regions. The 30 countries were used to create the geographical sub-regions based on their location in SSA and were categorized into Western Africa, Southern Africa, Eastern Africa, and Central Africa.

### Statistical analyses

Data for the study were analysed using Stata version 16. First, a forest plot was used to show the proportions of women who had autonomy to refuse risky sex across the 30 countries. Next, we examined the distribution of women’s autonomy in refusing risky sex across the explanatory variables. Thereafter, a chi-square test of independence was conducted to determine the explanatory variables that have significant associations with women’s ability to refuse risky sex. After this, we checked for a multicollinearity among the explanatory variables using the variance inflation factor (VIF) and the results showed no evidence of collinearity among the studied variables. Lastly, a multilevel binary logistic regression analysis, consisting of four models was conducted to examine the factors associated with women’s autonomy to refuse risky sex. The first model (Model O), which was an empty model with no explanatory variables showed the variance in the outcome variable attributed to the primary sampling unit (PSU). Model I had only the individual level variables and the outcome variable. Model II included only the contextual level variables and the outcome variable. The final model (Model III) included all the explanatory variables and the outcome variable. The regression analysis results were presented as adjusted odds ratios (aOR) with 95% confidence intervals (CIs). Additionally, fixed and random effects were added in all four models. Model O presented the random effects results and both fixed and random effects results were presented for Models I-III. The fixed effects results were presented as aORs with 95% CIs. Fixed effects represented the association between the explanatory variables and the outcome variable, whereas random effects showed the measure of variation in the outcome variable based on PSU (measured by Intra-Cluster Correlation [ICC]). The Akaike’s Information Criterion (AIC), which measures how well various models match the data, was used to evaluate model fitness. The multilevel regression models were run with Stata’s "melogit" command. All the analyses were weighted to adjust for the complex sampling structure of the data. We restricted our analysis to complete cases; therefore, all missing values were dropped.

### Ethical consideration

In this study, ethical clearance was not sought due to the public availability of the DHS dataset. The datasets were obtained from the MEASURE DHS after registration and approval were given for its usage. Detailed information about the DHS data usage and ethical standards are available at http://goo.gl/ny8T6X.

## Results

### Proportion of women’s autonomy to refuse sex in sub-Saharan Africa

**Fig 1:**
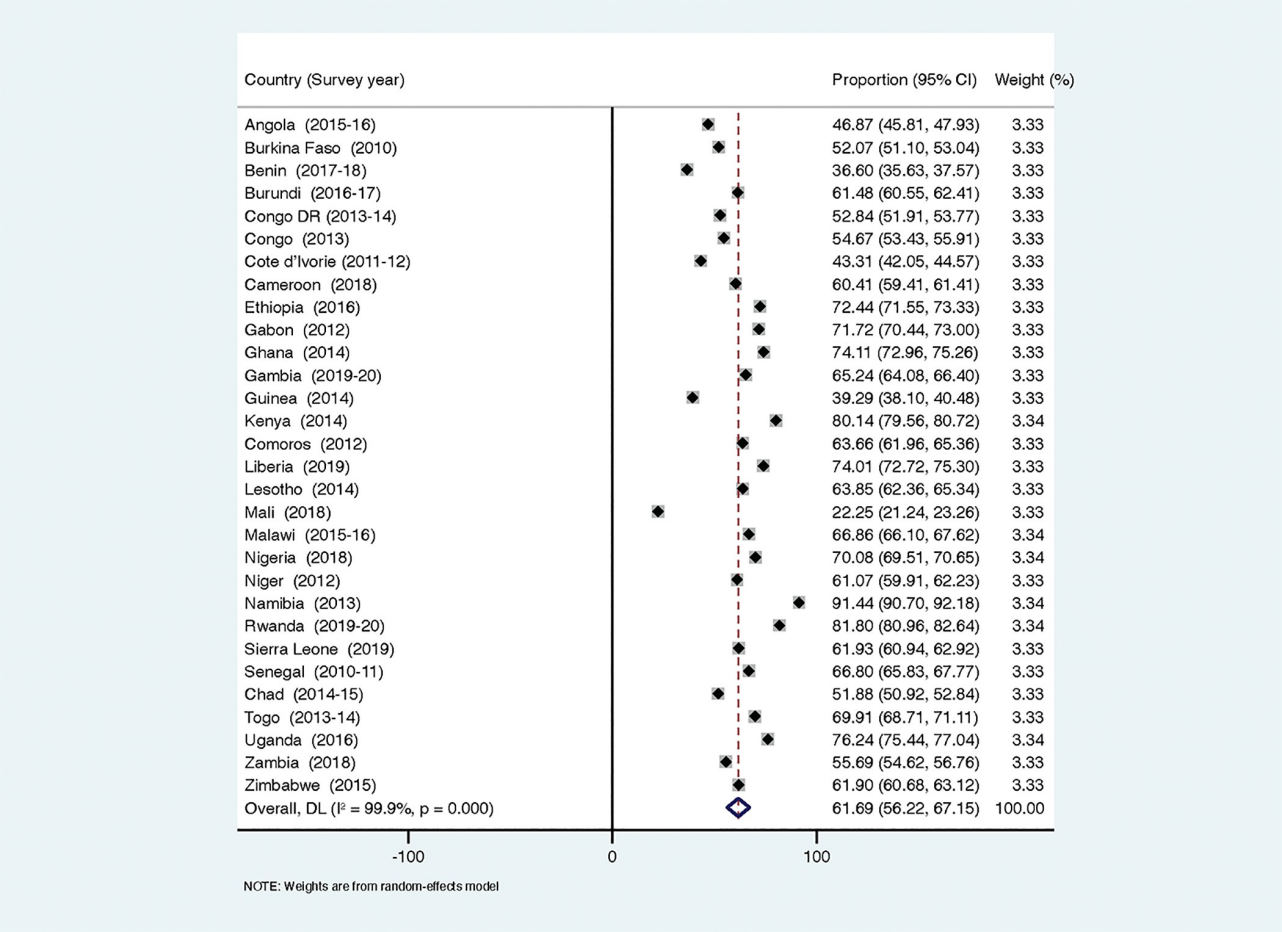
Proportion of women in sub-Saharan Africa who can refuse sex if their partners have other women.

Fig 1 shows the proportion of women who refused risky sex when their partners had other women in SSA. We found that 61.69% (95% CI: 56.22–67.15) of the women were autonomous in refusing sex if their partners had other women and this was highest in Namibia (91.44% [95% CI: 90.77–92.18]) and lowest in Mali (22.25% [95% CI: 21.24–23.26]).

### Distribution of women who can refused sex if their partners have other women across the explanatory variables

Table 2 shows the distribution of women who can refused sex if their partners have other women across the explanatory variables. It was observed that adolescents aged 15–19 had the lowest prevalence (59.4%) of refusing risky sex. The results further showed that women with no education (53.9%), those whose partners have no formal education (53.5%), women not working (59.8%), women whose partners were also not working (57.1%), those in the poorest wealth quintile (57.6%), and those in rural areas (60.7%) had the lowest proportion reporting an ability to refuse sex when their partners have other women. Women who had their first sex below 18 years had the lowest prevalence in terms of refusing risky sex (61.6%). Women who were not exposed to the mass media (56.5%) and those in Western Africa had the lowest proportion reporting an ability to refuse sex if their partners have other women. The chi-square analysis showed statistically significant differences for all the explanatory variables at p<0.001.

**Table 2:** Distribution of women who can refused sex if their partners have other women in sub-Saharan Africa.

Variables	Weighted frequency (n)	Weighted percentage (%)	Women’s autonomy to refuse risky sex	p-value
**Age**				<0.001
15–19	16293	6.3	59.4	
20–24	43793	16.8	62.0	
25–29	55871	21.5	63.1	
30–34	48576	18.7	63.4	
35–39	41602	16.0	63.2	
40–44	30161	11.6	63.3	
45–49	23727	9.1	62.7	
**Education**				<0.001
No education	100850	38.8	53.9	
Primary	82111	31.6	66.0	
Secondary	65008	25.0	68.9	
Higher	12056	4.6	78.8	
**Employment**				<0.001
Not working	65292	25.1	59.8	
Work	194733	74.9	63.7	
**Wealth**				<0.001
Poorest	51093	19.7	57.6	
Poorer	52622	20.2	60.0	
Middle	51412	19.8	62.5	
Richer	52310	20.1	64.2	
Richest	52589	20.2	69.2	
**Partner’s education**				<0.001
No education	86895	33.4	53.5	
Primary	70456	27.1	65.8	
Secondary	79465	30.6	66.7	
Higher	23209	8.9	73.6	
**Partner’s employment**				<0.001
Not Working	10598	4.1	57.1	
Working	249427	95.9	63.0	
**Age at first sex**				<0.001
Below 18 years	161869	62.3	61.6	
18 years and above	98156	37.7	64.7	
**Residence**				<0.001
Urban	93077	35.8	66.4	
Rural	166948	64.2	60.7	
**Mass media**				<0.001
No	85875	33.0	56.5	
Yes	174150	67.0	65.7	
**Geographic sub-regions**				<0.001
Western	103459	39.8	57.3	
Eastern	100512	38.7	70.4	
Central	39179	15.0	57.5	
Southern	16876	6.5	62.1	

### Predictors of women’s autonomy to refuse sex if partners have other women in sub-Saharan Africa

Table 3 shows the predictors of women’s autonomy to refuse sex if partners have other women in SSA. In terms of age, women aged 20–24, 25–29, 30–34, 35–39, 40–44, and 45–49 had varying odds of indicating their ability to refuse sex compared to those aged 15–19, with the highest odds observed for women aged 40–44 (aOR = 1.08; 95% CI = 1.03–1.20). The odds of refusing risky sex was higher among women with higher education (aOR = 1.88; 95% CI = 1.78–1.46) compared to those with no formal education. Employment status was also a significant predictor, with working women having higher odds of sex refusal compared to non-working women (aOR = 1.16; 95% CI = 1.13–1.18). Women whose partners had primary, secondary, or higher education exhibited higher odds of indicating their ability to refuse sex compared to those whose partners had no education, with the highest odds observed for partners with primary education (aOR = 1.72; 95% CI = 1.14–1.20). Similarly, women whose partners were working had higher odds of sex refusal compared to those whose partners were not working (aOR = 1.20; 95% CI = 1.15–1.25). Compared to women who had their first sex before age 18, there was a significantly higher likelihood of refusing risky sex among those who had their first sex at age 18 and above (aOR = 1.12; 95%CI = 1.10–1.14). Having exposure to the mass media (aOR = 1.24; 95%CI = 1.21–1.26) was associated with higher odds of refusing risky sex. Residing in rural areas (aOR = 0.95; 95%CI = 0.93–0.97) and belonging to the richer wealth quintile (aOR = 0.96; 95%CI = 0.93–0.99) were associated with lower likelihood of refusing risky sex compared to those living in urban areas and those in the poorest wealth quintile, respectively. Women in Eastern Africa (aOR = 1.52; 95%CI = 1.49–1.56) and Southern Africa (aOR = 1.25; 95% CI = 1.20–1.29) also had the highest odds of refusing risky sex compared to those in Western Africa.

**Table 3:** Predictors of sex refusal if partners have other women in sub-Sahara Africa.

Variables	Model O	Model I [aOR (95% CI)]	Model II [aOR (95% CI)]	Model III [aOR (95% CI)]
**Age**				
15–19		Ref (1.0)		Ref (1.0)
20–24		1.04 (1.00–1.08)		1.02 (0.98–1.05)
25–29		1.05** (1.01–1.09)		1.02 (0.99–1.06)
30–34		1.09*** (1.05–1.13)		1.05* (1.01–1.09)
35–39		1.10*** (1.06–1.15)		1.06** (1.02–1.10)
40–44		1.12*** (1.08–1.17)		1.08*** (1.03–1.20)
45–49		1.10*** (1.05–1.14)		1.06* (1.01–1.10)
**Education**				
No education		Ref (1.0)		Ref (1.0)
Primary		1.39*** (1.36–1.42)		1.28*** (1.25–1.31)
Secondary		1.51*** (1.47–1.56)		1.41***(1.37–1.46)
Higher		2.12*** (2.00–2.24)		1.88***(1.78–1.46)
**Employment**				
Not working		Ref (1.0)		Ref (1.0)
Working		1.07*** (1.05–1.09)		1.16*** (1.13–1.18)
**Partner’s education**				
No education		Ref (1.0)		Ref (1.0)
Primary		1.29*** (1.26–1.32)		1.72*** (1.14–1.20)
Secondary		1.20*** (1.17–1.23)		1.18*** (1.14–1.21)
Higher		1.35*** (1.30–1.41)		1.31*** (1.26–1.37)
**Partner’s employment**				
Not Working		Ref (1.0)		Ref (1.0)
Working		1.17*** (1.12–1.21)		1.20*** (1.15–1.25)
**Age at first sex**				
Below 18 years		Ref (1.0)		Ref (1.0)
18 years and above		1.09*** (1.07–1.11)		1.12*** (1.10–1.14)1
**Mass media**				
No		Ref (1.0)		Ref (1.0)
Yes		1.22*** (1.20–1.24)		1.24*** (1.21–1.26)
**Wealth**				
Poorest			Ref (1.0)	Ref (1.0)
Poorer			1.06*** (1.04–1.09)	0.99 (0.97–1.02)
Middle			1.10*** (1.07–1.13)	0.97 (0.94–1.00)
Richer			1.15*** (1.22–1.19)	0.96** (0.93–0.99)
Richest			1.53*** (1.29–1.37)	0.97 (0.94–1.00)
**Residence**				
Urban			Ref (1.0)	Ref (1.0)
Rural			0.87*** (0.85–0.89)	0.95*** (0.93–0.97)
**Africa**				
West			Ref (1.0)	Ref (1.0)
East			1.62*** (1.59–1.65)	1.52***(1.49–1.56)
Central			1.00 (0.98–1.03)	0.96*** (0.93–0.98)
South			1.25*** (1.21–1.29)	1.25*** (1.20–1.29)
**Random effect results**				
PSU variance	0.15 (0.13–0.17)	0.12 (0.11–0.14)	0.12 (0.11–0.14)	0.11 (0.09–0.13)
ICC	0.0426638	0.0355608	0.0362633	0.0319767
Wald chi-square		5800.67***	3985.39***	7479.62***
LR test	2069.99 (X = 0.000)	1843.43 (X = 0.000)	1697.07 (X = 0.000)	1619.86 (X = 0.000)
Model fitness				
Log-likelihood	-168763	-157671.36	-166719.88	-156750.32
AIC	337530	315378.7	333459.8	313552.6
N	260025	260025	260025	260025
Number of groups	1612	1612	1612	1612

*p<0.05

** p<0.01

*** p<0.001; aOR = Adjusted Odds Ratio; CI = Confidence Interval, Reference (1.0) = Reference category; PSU = Primary Sampling Unit; ICC = Intra-Class Correlation; AIC = Akaike’s Information Criterion.

## Discussion

This study examined the prevalence of women’s autonomy to refuse risky sex and its associated factors in SSA. Our findings indicate that more than half of women in SSA were autonomous in refusing risky sex. The estimated proportion is similar to what has been reported in Ethiopia (69.5%) [5]. However, it is lower than the estimated proportion reported in Bangladesh (92%) [20]. Perhaps the vast difference between our findings and that of Jesmin et al. [20] could be explained by the point that they used STIs as the condition to refuse sex while our study was premised on refusing sex when one’s partner has been sexually involved with other women. Despite the moderately high prevalence across SSA, there were substantial sub-regional and country differences. Namibia reported the highest prevalence of women’s autonomy to refuse risky sex while Mali had the lowest prevalence. Moreover, at the sub-regional level, the Eastern and Southern parts of SSA had higher likelihood of refusing risky sex, while women in the Central part had significantly lower odds of refusing risky sex when compared to those from Western Africa. Perhaps, these country and regional variations may be a reflection of the level of commitment that the respective countries have made towards empowering women to be autonomous in healthcare decision-making such as refusing risky sex.

It is indicative that the likelihood of refusing risky sex increases with age. The result is in contrast to the findings of a previous study [5] that found the likelihood of refusing risky sex to be significantly lower among older women in comparison to adolescent girls and young women; this was justified by the assumption that “older age is associated with decreases in self-esteem”, hence, reducing their autonomy and confidence to refuse risky sex [21]. However, our study suggests that older women are more likely to refuse risky sex compared to adolescent girls (i.e., 15–19 years). This finding is supported by a related study conducted in SSA [3] which found that older women are more capable of negotiating for safe sex. Possibly, adolescent girls often lack the knowledge and experience about safe sex, and are more likely to be easily swayed by curiosity and peers to practice risky sexual activities. The same cannot be said about women of older age, who may have accumulated a wealth of knowledge and experience to inform their autonomy to refuse risky sex.

Education was another significant factor that predicted the likelihood of women’s autonomy to refuse risky sex. Higher educational attainment of the woman and her partner was associated with higher odds of having the autonomy to refuse risky sex. Similar findings have been reported in SSA [3] and Ethiopia [5]. Education provides women with accurate and comprehensive information concerning STIs and HIV/AIDS; this knowledge enables women to understand the importance of adopting healthy sexual behaviours [22], and thus, enhance their autonomy to refuse risky sex. Having high educational attainment also enables male partners to be receptive to the demands of their partners/spouses regarding safe sex practices. Male partners also have a high probability of understanding the dangers of practicing risky sex behaviours, thereby enabling their partners to be assertive to be autonomous in their decision to refuse risky sex.

The study also revealed that women who were working had a higher likelihood to be autonomous in refusing risky sex than their counterparts who were not in any sort of employment. Likewise, women who were in union with male partners who were working had higher odds of refusing risky sex. Seidu et al. [3] and De Coninck et al. [23] have also found similar direction of association between employment status and women’s autonomy to refuse risky sex. Women were employed might have gained financial and psychological independence that emboldens them to be autonomous to refuse risky sex and opt for safer sex practices.

Women’s autonomy to refuse risky sex was positively associated with exposure to the mass media. That is, women who had access to mass media were more likely to refuse risky sex as compared to those who had no exposure to mass media platforms. Similar findings have been reported in previous studies [3,24]. This might be due to the assertion that mass media plays a quintessential role in promoting awareness and increasing women’s knowledge about the consequences of risky sexual behaviour. Consequently, the knowledge gained could enhance women’s autonomy to refuse risky sex. Thus, highlighting the significance of the media in advocating for women’s autonomy and safer sex practices.

Compared to women who had their first sex before age 18, those who had their first sex at age 18 and above were more likely to refuse risky sex. This finding confirms that of previous studies [25,26] where early sexual debut was associated with multiple sexual behaviours including the capacity to refuse risky sex. It is possible that women who delayed their sexual debut until after the age of 18 might have had more time for personal development, education, and awareness about sexual health, which could have contributed to their increased autonomy in refusing risky sex.

### Implications for policy and practice

Results from the study highlights a need to leverage media platforms to promote women’s empowerment, sexual health education, and safer sex practices. Leveraging the mass media can effectively reach a wider audience and provide essential information to empower women in making informed decisions about their sexual health. The findings regarding work status and women’s autonomy to refuse risky sex underscores a need to create more economic and livelihood interventions for women so that they can be economically autonomous to insist on safer sex practices. Also, sub-Saharan African countries must intensify efforts to enhance the assertiveness of adolescent girls on issues regarding safe sex practices.

### Strengths and limitations

The use of a nationally representative dataset, the DHS, for analysis is the study’s main strength. Furthermore, focusing on 30 countries in SSA allows for a better understanding of the differences between countries in terms of women’s autonomy to refuse risky sex. We recognise that this study has limitations, and as such, interpretations and inferences drawn from the data must consider these limitations. Because the current study used a cross-sectional methodology, causal inferences cannot be established between the associated factors of women’s autonomy to refuse risky sex. Furthermore, the variable for risky sex was self-reported, making it susceptible to social desirability bias.

## Conclusion

The study has shown that women’s autonomy to refuse risky sex was moderately high in SSA. The associated factors of women’s autonomy to refuse risky sex were age, woman’s educational attainment and employment status, partner’s educational attainment and employment status, exposure to the mass media, and region of residence. Advocacy to improve women’s autonomy to refuse risky sex must leverage the mass media as it emerged as a significant factor. Policies and intervention to enhance women’s autonomy must also target high-risk sub-populations which constitutes adolescent girls, those with no formal education, and those without employment. Also, inferring from the association between employment status and women’s autonomy to refuse risky sex, sub-Saharan African countries must invest in empowering women with economic livelihood activities to enhance their level of autonomy and independence.
